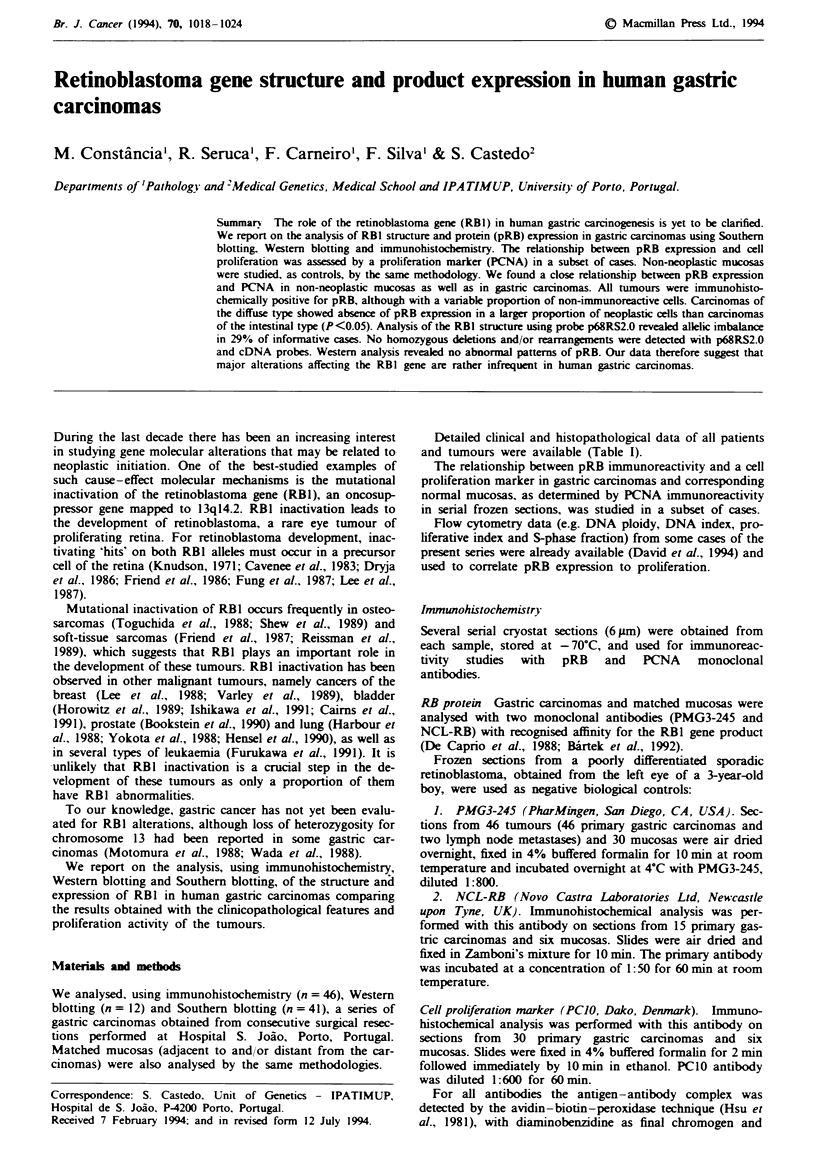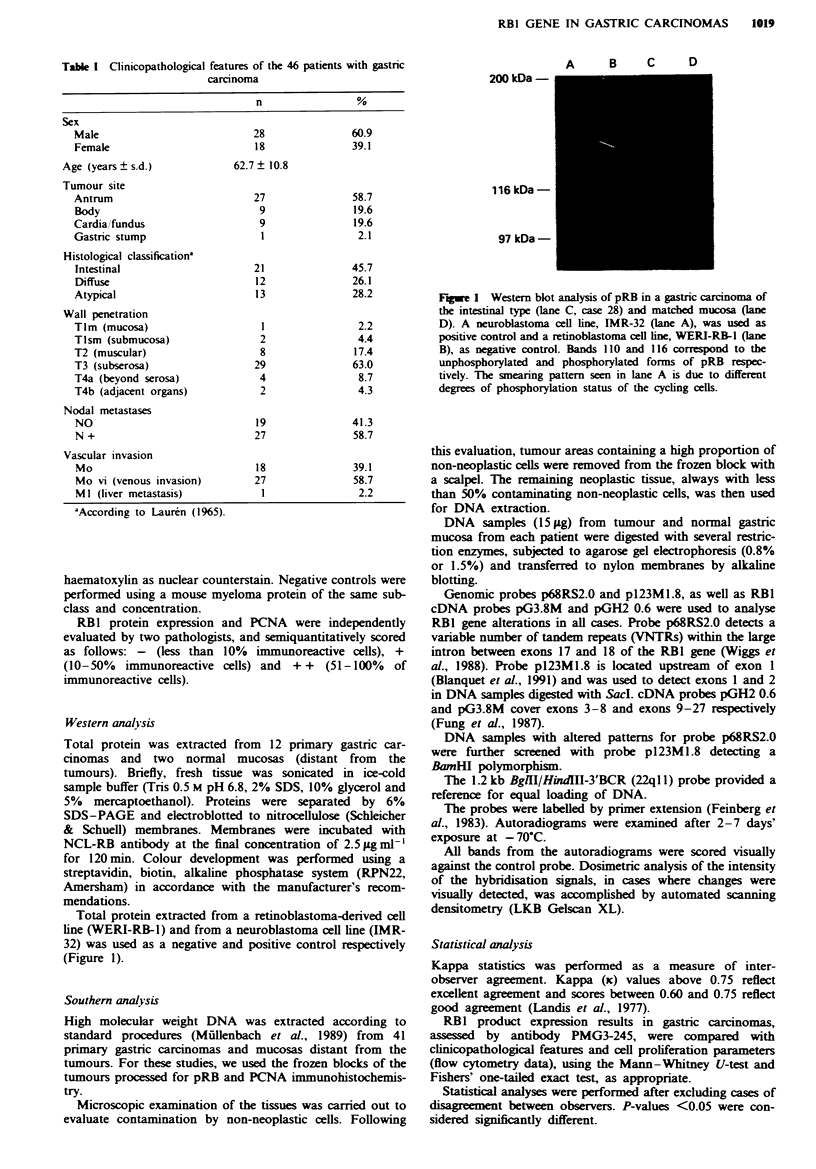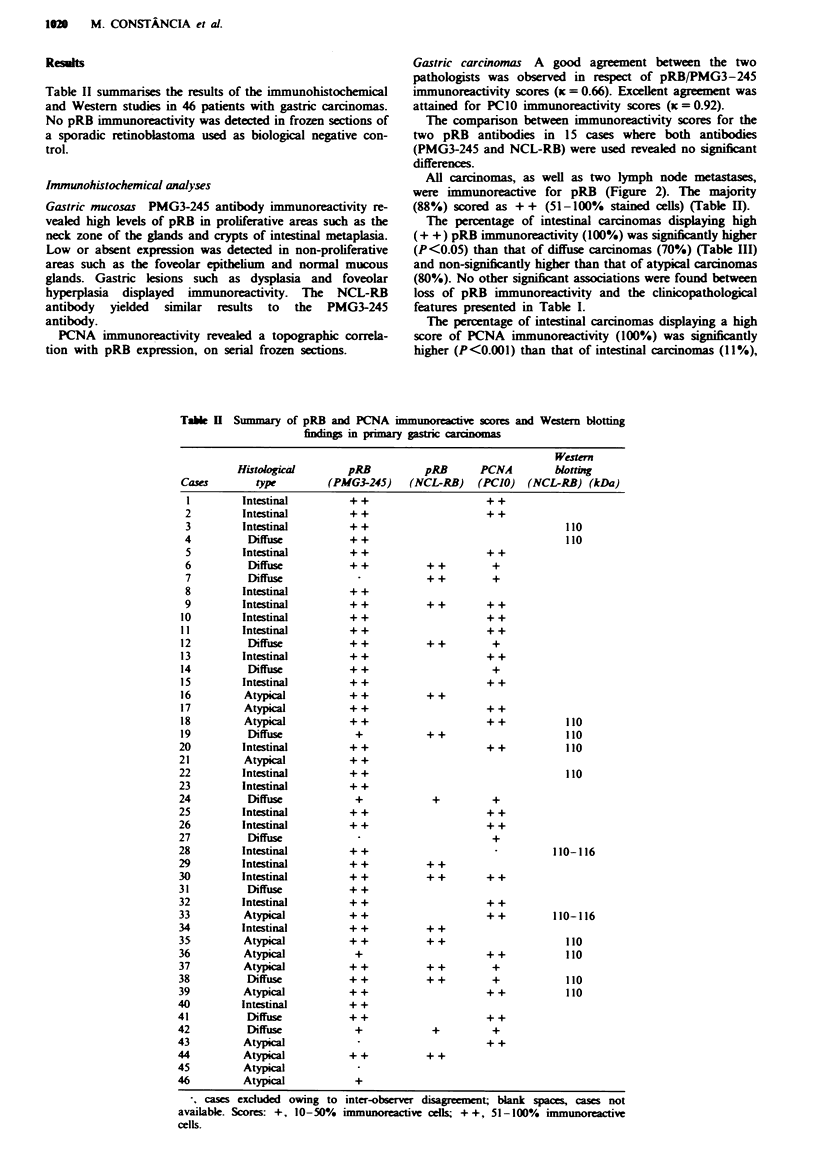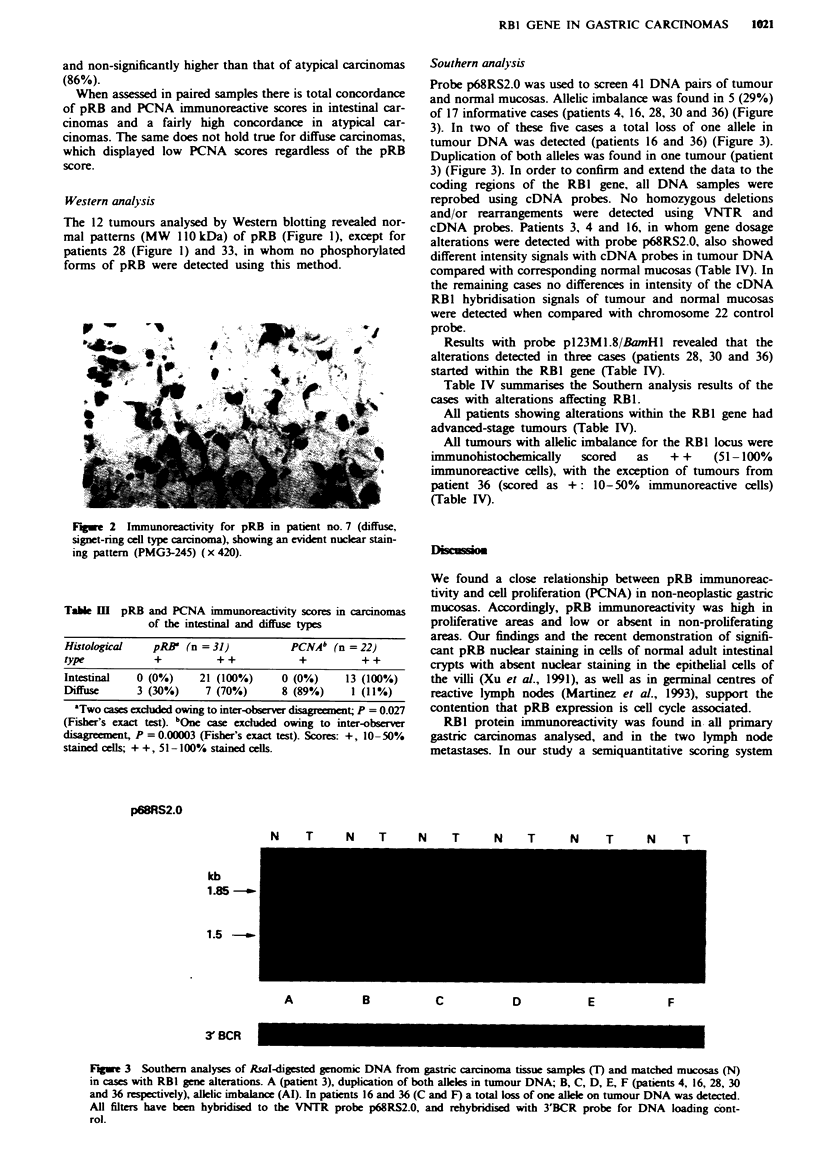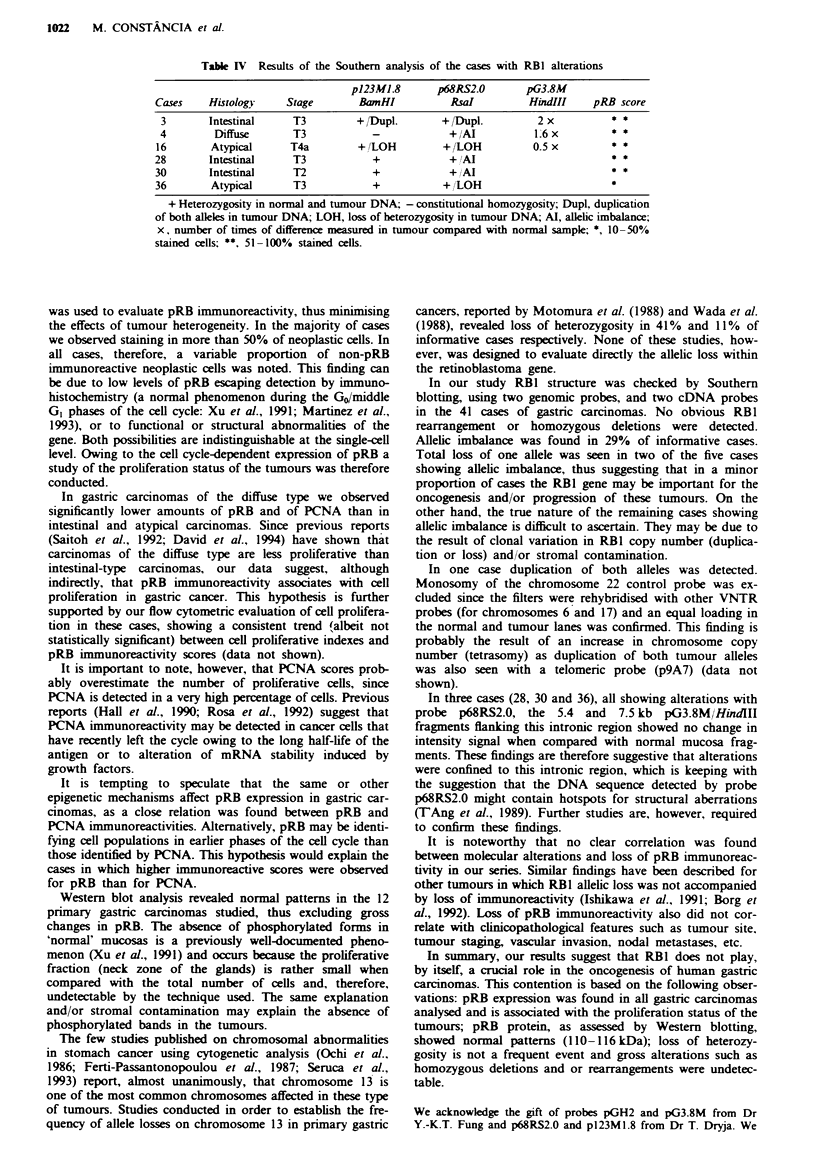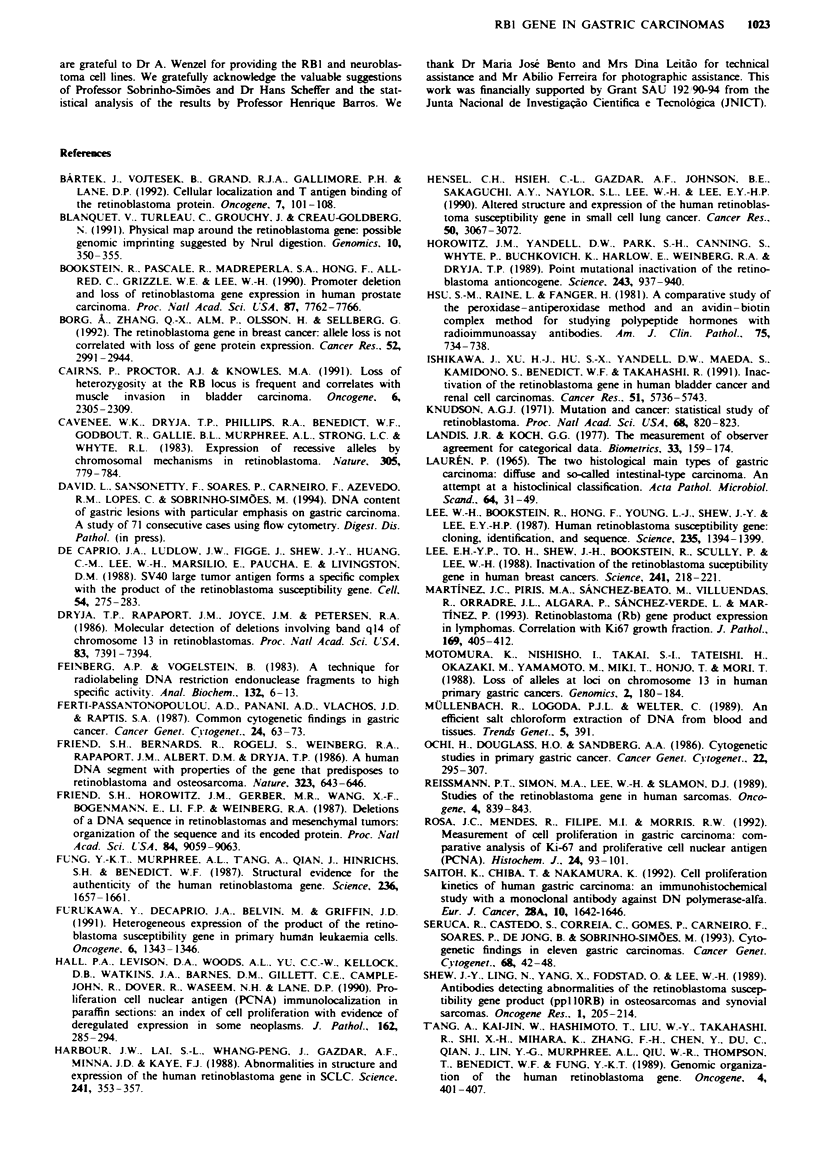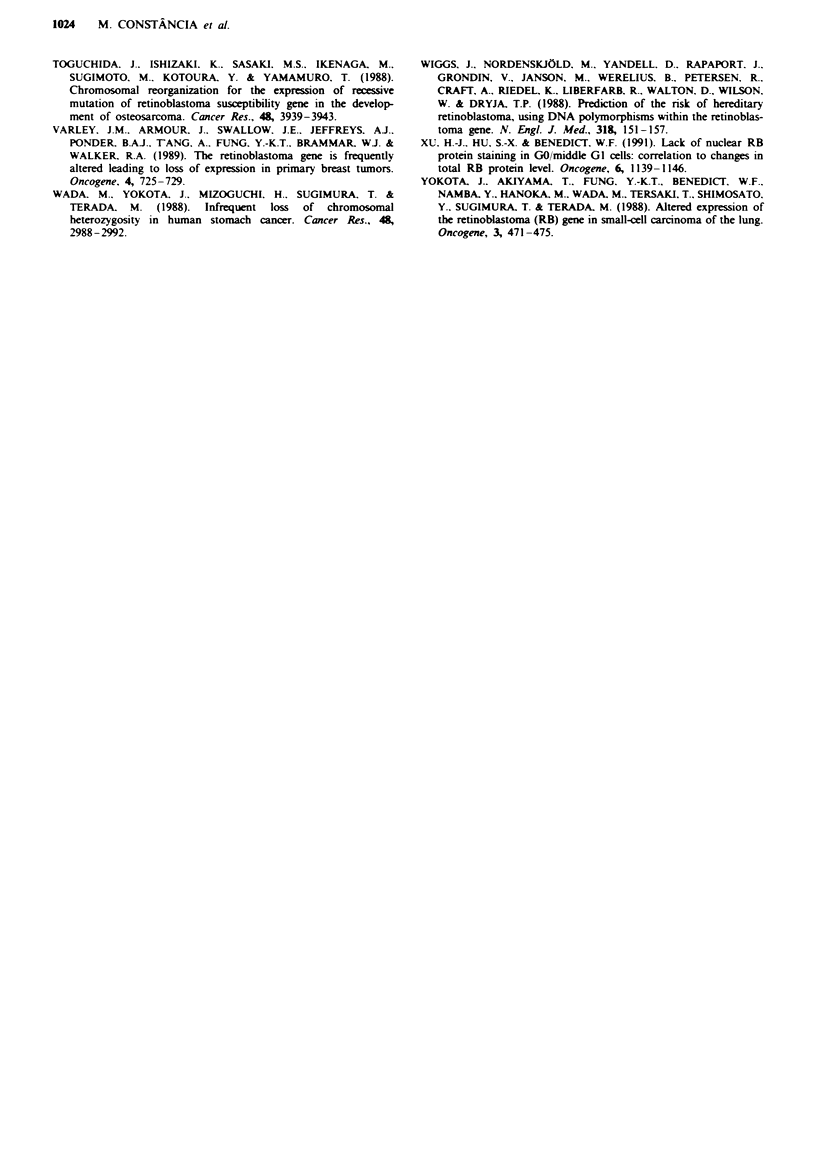# Retinoblastoma gene structure and product expression in human gastric carcinomas.

**DOI:** 10.1038/bjc.1994.441

**Published:** 1994-11

**Authors:** M. Constância, R. Seruca, F. Carneiro, F. Silva, S. Castedo

**Affiliations:** Department of Pathology, Medical School, University of Porto, Portugal.

## Abstract

**Images:**


Br. J. Cancer (1994), 70, 1018-1024 © Macmillan Press Ltd., 1994

### Retinoblastoma gene structure and product expression in human gastric carcinomas

M. Constância[1], R. Seruca[1], F. Carneiro[1], F. Silva[1] & S. Castedo[2]

*Departments of [1]Pathology and [2]Medical Genetics, Medical School and IPATIMUP, University of Porto, Portugal.*

**Summary** The role of the retinoblastoma gene (RB1) in human gastric carcinogenesis is yet to be clarified. We report on the analysis of RB1 structure and protein (pRB) expression in gastric carcinomas using Southern blotting, Western blotting and immunohistochemistry. The relationship between pRB expression and cell proliferation was assessed by a proliferation marker (PCNA) in a subset of cases. Non-neoplastic mucosas were studied, as controls, by the same methodology. We found a close relationship between pRB expression and PCNA in non-neoplastic mucosas as well as in gastric carcinomas. All tumours were immunohistochemically positive for pRB, although with a variable proportion of non-immunoreactive cells. Carcinomas of the diffuse type showed absence of pRB expression in a larger proportion of neoplastic cells than carcinomas of the intestinal type ($P<0.05$). Analysis of the RB1 structure using probe p68RS2.0 revealed allelic imbalance in 29% of informative cases. No homozygous deletions and/or rearrangements were detected with p68RS2.0 and cDNA probes. Western analysis revealed no abnormal patterns of pRB. Our data therefore suggest that major alterations affecting the RB1 gene are rather infrequent in human gastric carcinomas.

During the last decade there has been an increasing interest in studying gene molecular alterations that may be related to neoplastic initiation. One of the best-studied examples of such cause-effect molecular mechanisms is the mutational inactivation of the retinoblastoma gene (RB1), an oncosuppressor gene mapped to 13q14.2. RB1 inactivation leads to the development of retinoblastoma, a rare eye tumour of proliferating retina. For retinoblastoma development, inactivating 'hits' on both RB1 alleles must occur in a precursor cell of the retina (Knudson, 1971; Cavenee *et al.*, 1983; Dryja *et al.*, 1986; Friend *et al.*, 1986; Fung *et al.*, 1987; Lee *et al.*, 1987).

Mutational inactivation of RB1 occurs frequently in osteosarcomas (Toguchida *et al.*, 1988; Shew *et al.*, 1989) and soft-tissue sarcomas (Friend *et al.*, 1987; Reissman *et al.*, 1989), which suggests that RB1 plays an important role in the development of these tumours. RB1 inactivation has been observed in other malignant tumours, namely cancers of the breast (Lee *et al.*, 1988; Varley *et al.*, 1989), bladder (Horowitz *et al.*, 1989; Ishikawa *et al.*, 1991; Cairns *et al.*, 1991), prostate (Bookstein *et al.*, 1990) and lung (Harbour *et al.*, 1988; Yokota *et al.*, 1988; Hensel *et al.*, 1990), as well as in several types of leukaemia (Furukawa *et al.*, 1991). It is unlikely that RB1 inactivation is a crucial step in the development of these tumours as only a proportion of them have RB1 abnormalities.

To our knowledge, gastric cancer has not yet been evaluated for RB1 alterations, although loss of heterozygosity for chromosome 13 had been reported in some gastric carcinomas (Motomura *et al.*, 1988; Wada *et al.*, 1988).

We report on the analysis, using immunohistochemistry, Western blotting and Southern blotting, of the structure and expression of RB1 in human gastric carcinomas comparing the results obtained with the clinicopathological features and proliferation activity of the tumours.

### Materials and methods

We analysed, using immunohistochemistry ($n = 46$), Western blotting ($n = 12$) and Southern blotting ($n = 41$), a series of gastric carcinomas obtained from consecutive surgical resections performed at Hospital S. João, Porto, Portugal. Matched mucosas (adjacent to and/or distant from the carcinomas) were also analysed by the same methodologies.

Correspondence: S. Castedo, Unit of Genetics - IPATIMUP, Hospital de S. João, P-4200 Porto, Portugal.
Received 7 February 1994; and in revised form 12 July 1994.

Detailed clinical and histopathological data of all patients and tumours were available (Table I).

The relationship between pRB immunoreactivity and a cell proliferation marker in gastric carcinomas and corresponding normal mucosas, as determined by PCNA immunoreactivity in serial frozen sections, was studied in a subset of cases.

Flow cytometry data (e.g. DNA ploidy, DNA index, proliferative index and S-phase fraction) from some cases of the present series were already available (David *et al.*, 1994) and used to correlate pRB expression to proliferation.

### *Immunohistochemistry*

Several serial cryostat sections (6 µm) were obtained from each sample, stored at −70°C, and used for immunoreactivity studies with pRB and PCNA monoclonal antibodies.

*RB protein* Gastric carcinomas and matched mucosas were analysed with two monoclonal antibodies (PMG3-245 and NCL-RB) with recognised affinity for the RB1 gene product (De Caprio *et al.*, 1988; Bártek *et al.*, 1992).

Frozen sections from a poorly differentiated sporadic retinoblastoma, obtained from the left eye of a 3-year-old boy, were used as negative biological controls:

1. *PMG3-245 (PharMingen, San Diego, CA, USA).* Sections from 46 tumours (46 primary gastric carcinomas and two lymph node metastases) and 30 mucosas were air dried overnight, fixed in 4% buffered formalin for 10 min at room temperature and incubated overnight at 4°C with PMG3-245, diluted 1:800.
2. *NCL-RB (Novo Castra Laboratories Ltd, Newcastle upon Tyne, UK).* Immunohistochemical analysis was performed with this antibody on sections from 15 primary gastric carcinomas and six mucosas. Slides were air dried and fixed in Zamboni's mixture for 10 min. The primary antibody was incubated at a concentration of 1:50 for 60 min at room temperature.

*Cell proliferation marker (PC10, Dako, Denmark).* Immunohistochemical analysis was performed with this antibody on sections from 30 primary gastric carcinomas and six mucosas. Slides were fixed in 4% buffered formalin for 2 min followed immediately by 10 min in ethanol. PC10 antibody was diluted 1:600 for 60 min.

For all antibodies the antigen-antibody complex was detected by the avidin-biotin-peroxidase technique (Hsu *et al.*, 1981), with diaminobenzidine as final chromogen and

Table I Clinicopathological features of the 46 patients with gastric carcinoma

| | n | % |
|---|---|---|
| Sex | | |
| Male | 28 | 60.9 |
| Female | 18 | 39.1 |
| Age (years ± s.d.) | 62.7 ± 10.8 | |
| Tumour site | | |
| Antrum | 27 | 58.7 |
| Body | 9 | 19.6 |
| Cardia/fundus | 9 | 19.6 |
| Gastric stump | 1 | 2.1 |
| Histological classification[a] | | |
| Intestinal | 21 | 45.7 |
| Diffuse | 12 | 26.1 |
| Atypical | 13 | 28.2 |
| Wall penetration | | |
| T1m (mucosa) | 1 | 2.2 |
| T1sm (submucosa) | 2 | 4.4 |
| T2 (muscular) | 8 | 17.4 |
| T3 (subserosa) | 29 | 63.0 |
| T4a (beyond serosa) | 4 | 8.7 |
| T4b (adjacent organs) | 2 | 4.3 |
| Nodal metastases | | |
| NO | 19 | 41.3 |
| N + | 27 | 58.7 |
| Vascular invasion | | |
| Mo | 18 | 39.1 |
| Mo vi (venous invasion) | 27 | 58.7 |
| M1 (liver metastasis) | 1 | 2.2 |

[a]According to Laurén (1965).

**Figure 1** Western blot analysis of pRB in a gastric carcinoma of the intestinal type (lane C, case 28) and matched mucosa (lane D). A neuroblastoma cell line, IMR-32 (lane A), was used as positive control and a retinoblastoma cell line, WERI-RB-1 (lane B), as negative control. Bands 110 and 116 correspond to the unphosphorylated and phosphorylated forms of pRB respectively. The smearing pattern seen in lane A is due to different degrees of phosphorylation status of the cycling cells.

haematoxylin as nuclear counterstain. Negative controls were performed using a mouse myeloma protein of the same subclass and concentration.

RB1 protein expression and PCNA were independently evaluated by two pathologists, and semiquantitatively scored as follows: − (less than 10% immunoreactive cells), + (10–50% immunoreactive cells) and + + (51–100% of immunoreactive cells).

*Western analysis*

Total protein was extracted from 12 primary gastric carcinomas and two normal mucosas (distant from the tumours). Briefly, fresh tissue was sonicated in ice-cold sample buffer (Tris 0.5 M pH 6.8, 2% SDS, 10% glycerol and 5% mercaptoethanol). Proteins were separated by 6% SDS–PAGE and electroblotted to nitrocellulose (Schleicher & Schuell) membranes. Membranes were incubated with NCL-RB antibody at the final concentration of 2.5 $\mu g\ ml^{-1}$ for 120 min. Colour development was performed using a streptavidin, biotin, alkaline phosphatase system (RPN22, Amersham) in accordance with the manufacturer's recommendations.

Total protein extracted from a retinoblastoma-derived cell line (WERI-RB-1) and from a neuroblastoma cell line (IMR-32) was used as a negative and positive control respectively (Figure 1).

*Southern analysis*

High molecular weight DNA was extracted according to standard procedures (Müllenbach *et al*., 1989) from 41 primary gastric carcinomas and mucosas distant from the tumours. For these studies, we used the frozen blocks of the tumours processed for pRB and PCNA immunohistochemistry.

Microscopic examination of the tissues was carried out to evaluate contamination by non-neoplastic cells. Following this evaluation, tumour areas containing a high proportion of non-neoplastic cells were removed from the frozen block with a scalpel. The remaining neoplastic tissue, always with less than 50% contaminating non-neoplastic cells, was then used for DNA extraction.

DNA samples (15 μg) from tumour and normal gastric mucosa from each patient were digested with several restriction enzymes, subjected to agarose gel electrophoresis (0.8% or 1.5%) and transferred to nylon membranes by alkaline blotting.

Genomic probes p68RS2.0 and p123M1.8, as well as RB1 cDNA probes pG3.8M and pGH2 0.6 were used to analyse RB1 gene alterations in all cases. Probe p68RS2.0 detects a variable number of tandem repeats (VNTRs) within the large intron between exons 17 and 18 of the RB1 gene (Wiggs *et al*., 1988). Probe p123M1.8 is located upstream of exon 1 (Blanquet *et al*., 1991) and was used to detect exons 1 and 2 in DNA samples digested with *Sac*I. cDNA probes pGH2 0.6 and pG3.8M cover exons 3–8 and exons 9–27 respectively (Fung *et al*., 1987).

DNA samples with altered patterns for probe p68RS2.0 were further screened with probe p123M1.8 detecting a *Bam*HI polymorphism.

The 1.2 kb *Bgl*II/*Hind*III-3'BCR (22q11) probe provided a reference for equal loading of DNA.

The probes were labelled by primer extension (Feinberg *et al*., 1983). Autoradiograms were examined after 2–7 days' exposure at −70°C.

All bands from the autoradiograms were scored visually against the control probe. Dosimetric analysis of the intensity of the hybridisation signals, in cases where changes were visually detected, was accomplished by automated scanning densitometry (LKB Gelscan XL).

*Statistical analysis*

Kappa statistics was performed as a measure of interobserver agreement. Kappa (κ) values above 0.75 reflect excellent agreement and scores between 0.60 and 0.75 reflect good agreement (Landis *et al*., 1977).

RB1 product expression results in gastric carcinomas, assessed by antibody PMG3-245, were compared with clinicopathological features and cell proliferation parameters (flow cytometry data), using the Mann–Whitney *U*-test and Fishers' one-tailed exact test, as appropriate.

Statistical analyses were performed after excluding cases of disagreement between observers. *P*-values $<0.05$ were considered significantly different.

### Results

Table II summarises the results of the immunohistochemical and Western studies in 46 patients with gastric carcinomas. No pRB immunoreactivity was detected in frozen sections of a sporadic retinoblastoma used as biological negative control.

### *Immunohistochemical analyses*

*Gastric mucosas* PMG3-245 antibody immunoreactivity revealed high levels of pRB in proliferative areas such as the neck zone of the glands and crypts of intestinal metaplasia. Low or absent expression was detected in non-proliferative areas such as the foveolar epithelium and normal mucous glands. Gastric lesions such as dysplasia and foveolar hyperplasia displayed immunoreactivity. The NCL-RB antibody yielded similar results to the PMG3-245 antibody.

PCNA immunoreactivity revealed a topographic correlation with pRB expression, on serial frozen sections.

*Gastric carcinomas* A good agreement between the two pathologists was observed in respect of pRB/PMG3-245 immunoreactivity scores ($\kappa = 0.66$). Excellent agreement was attained for PC10 immunoreactivity scores ($\kappa = 0.92$).

The comparison between immunoreactivity scores for the two pRB antibodies in 15 cases where both antibodies (PMG3-245 and NCL-RB) were used revealed no significant differences.

All carcinomas, as well as two lymph node metastases, were immunoreactive for pRB (Figure 2). The majority (88%) scored as + + (51–100% stained cells) (Table II).

The percentage of intestinal carcinomas displaying high (+ +) pRB immunoreactivity (100%) was significantly higher ($P<0.05$) than that of diffuse carcinomas (70%) (Table III) and non-significantly higher than that of atypical carcinomas (80%). No other significant associations were found between loss of pRB immunoreactivity and the clinicopathological features presented in Table I.

The percentage of intestinal carcinomas displaying a high score of PCNA immunoreactivity (100%) was significantly higher ($P<0.001$) than that of intestinal carcinomas (11%),

**Table II** Summary of pRB and PCNA immunoreactive scores and Western blotting findings in primary gastric carcinomas

| *Cases* | *Histological type* | *pRB (PMG3-245)* | *pRB (NCL-RB)* | *PCNA (PC10)* | *Western blotting (NCL-RB) (kDa)* |
|---|---|---|---|---|---|
| 1 | Intestinal | + + | | + + | |
| 2 | Intestinal | + + | | + + | |
| 3 | Intestinal | + + | | | 110 |
| 4 | Diffuse | + + | | | 110 |
| 5 | Intestinal | + + | | + + | |
| 6 | Diffuse | + + | + + | + | |
| 7 | Diffuse | · | + + | + | |
| 8 | Intestinal | + + | | | |
| 9 | Intestinal | + + | + + | + + | |
| 10 | Intestinal | + + | | + + | |
| 11 | Intestinal | + + | | + + | |
| 12 | Diffuse | + + | + + | + | |
| 13 | Intestinal | + + | | + + | |
| 14 | Diffuse | + + | | + | |
| 15 | Intestinal | + + | | + + | |
| 16 | Atypical | + + | + + | | |
| 17 | Atypical | + + | | + + | |
| 18 | Atypical | + + | | + + | 110 |
| 19 | Diffuse | + | + + | | 110 |
| 20 | Intestinal | + + | | + + | 110 |
| 21 | Atypical | + + | | | |
| 22 | Intestinal | + + | | | 110 |
| 23 | Intestinal | + + | | | |
| 24 | Diffuse | + | + | + | |
| 25 | Intestinal | + + | | + + | |
| 26 | Intestinal | + + | | + + | |
| 27 | Diffuse | · | | + | |
| 28 | Intestinal | + + | | · | 110–116 |
| 29 | Intestinal | + + | + + | | |
| 30 | Intestinal | + + | + + | + + | |
| 31 | Diffuse | + + | | | |
| 32 | Intestinal | + + | | + + | |
| 33 | Atypical | + + | | + + | 110–116 |
| 34 | Intestinal | + + | + + | | |
| 35 | Atypical | + + | + + | | 110 |
| 36 | Atypical | + | | + + | 110 |
| 37 | Atypical | + + | + + | + | |
| 38 | Diffuse | + + | + + | + | 110 |
| 39 | Atypical | + + | | + + | 110 |
| 40 | Intestinal | + + | | | |
| 41 | Diffuse | + + | | + + | |
| 42 | Diffuse | + | + | + | |
| 43 | Atypical | · | | + + | |
| 44 | Atypical | + + | + + | | |
| 45 | Atypical | · | | | |
| 46 | Atypical | + | | | |

·, cases excluded owing to inter-observer disagreement; blank spaces, cases not available. Scores: +, 10–50% immunoreactive cells; + +, 51–100% immunoreactive cells.

and non-significantly higher than that of atypical carcinomas (86%).

When assessed in paired samples there is total concordance of pRB and PCNA immunoreactive scores in intestinal carcinomas and a fairly high concordance in atypical carcinomas. The same does not hold true for diffuse carcinomas, which displayed low PCNA scores regardless of the pRB score.

*Western analysis*

The 12 tumours analysed by Western blotting revealed normal patterns (MW 110 kDa) of pRB (Figure 1), except for patients 28 (Figure 1) and 33, in whom no phosphorylated forms of pRB were detected using this method.

**Figure 2** Immunoreactivity for pRB in patient no. 7 (diffuse, signet-ring cell type carcinoma), showing an evident nuclear staining pattern (PMG3-245) (× 420).

**Table III** pRB and PCNA immunoreactivity scores in carcinomas of the intestinal and diffuse types

| *Histological type* | *pRB*[a] (n = 31) + | ++ | *PCNA*[b] (n = 22) + | ++ |
|---|---|---|---|---|
| Intestinal | 0 (0%) | 21 (100%) | 0 (0%) | 13 (100%) |
| Diffuse | 3 (30%) | 7 (70%) | 8 (89%) | 1 (11%) |

[a]Two cases excluded owing to inter-observer disagreement; $P = 0.027$ (Fisher's exact test). [b]One case excluded owing to inter-observer disagreement, $P = 0.00003$ (Fisher's exact test). Scores: +, 10–50% stained cells; + +, 51–100% stained cells.

*Southern analysis*

Probe p68RS2.0 was used to screen 41 DNA pairs of tumour and normal mucosas. Allelic imbalance was found in 5 (29%) of 17 informative cases (patients 4, 16, 28, 30 and 36) (Figure 3). In two of these five cases a total loss of one allele in tumour DNA was detected (patients 16 and 36) (Figure 3). Duplication of both alleles was found in one tumour (patient 3) (Figure 3). In order to confirm and extend the data to the coding regions of the RB1 gene, all DNA samples were reprobed using cDNA probes. No homozygous deletions and/or rearrangements were detected using VNTR and cDNA probes. Patients 3, 4 and 16, in whom gene dosage alterations were detected with probe p68RS2.0, also showed different intensity signals with cDNA probes in tumour DNA compared with corresponding normal mucosas (Table IV). In the remaining cases no differences in intensity of the cDNA RB1 hybridisation signals of tumour and normal mucosas were detected when compared with chromosome 22 control probe.

Results with probe p123M1.8/*Bam*H1 revealed that the alterations detected in three cases (patients 28, 30 and 36) started within the RB1 gene (Table IV).

Table IV summarises the Southern analysis results of the cases with alterations affecting RB1.

All patients showing alterations within the RB1 gene had advanced-stage tumours (Table IV).

All tumours with allelic imbalance for the RB1 locus were immunohistochemically scored as + + (51–100% immunoreactive cells), with the exception of tumours from patient 36 (scored as +: 10–50% immunoreactive cells) (Table IV).

### Discussion

We found a close relationship between pRB immunoreactivity and cell proliferation (PCNA) in non-neoplastic gastric mucosas. Accordingly, pRB immunoreactivity was high in proliferative areas and low or absent in non-proliferating areas. Our findings and the recent demonstration of significant pRB nuclear staining in cells of normal adult intestinal crypts with absent nuclear staining in the epithelial cells of the villi (Xu *et al.*, 1991), as well as in germinal centres of reactive lymph nodes (Martinez *et al.*, 1993), support the contention that pRB expression is cell cycle associated.

RB1 protein immunoreactivity was found in all primary gastric carcinomas analysed, and in the two lymph node metastases. In our study a semiquantitative scoring system

**Figure 3** Southern analyses of *Rsa*I-digested genomic DNA from gastric carcinoma tissue samples (T) and matched mucosas (N) in cases with RB1 gene alterations. A (patient 3), duplication of both alleles in tumour DNA; B, C, D, E, F (patients 4, 16, 28, 30 and 36 respectively), allelic imbalance (AI). In patients 16 and 36 (C and F) a total loss of one allele on tumour DNA was detected. All filters have been hybridised to the VNTR probe p68RS2.0, and rehybridised with 3'BCR probe for DNA loading control.

Table IV Results of the Southern analysis of the cases with RB1 alterations

| Cases | Histology | Stage | p123M1.8 BamHI | p68RS2.0 RsaI | pG3.8M HindIII | pRB score |
|---|---|---|---|---|---|---|
| 3 | Intestinal | T3 | +/Dupl. | +/Dupl. | 2 × | * * |
| 4 | Diffuse | T3 | – | +/AI | 1.6 × | * * |
| 16 | Atypical | T4a | +/LOH | +/LOH | 0.5 × | * * |
| 28 | Intestinal | T3 | + | +/AI | | * * |
| 30 | Intestinal | T2 | + | +/AI | | * * |
| 36 | Atypical | T3 | + | +/LOH | | * |

+ Heterozygosity in normal and tumour DNA; – constitutional homozygosity; Dupl, duplication of both alleles in tumour DNA; LOH, loss of heterozygosity in tumour DNA; AI, allelic imbalance; ×, number of times of difference measured in tumour compared with normal sample; *, 10–50% stained cells; **, 51–100% stained cells.

was used to evaluate pRB immunoreactivity, thus minimising the effects of tumour heterogeneity. In the majority of cases we observed staining in more than 50% of neoplastic cells. In all cases, therefore, a variable proportion of non-pRB immunoreactive neoplastic cells was noted. This finding can be due to low levels of pRB escaping detection by immunohistochemistry (a normal phenomenon during the $G_0$/middle $G_1$ phases of the cell cycle: Xu *et al.*, 1991; Martinez *et al.*, 1993), or to functional or structural abnormalities of the gene. Both possibilities are indistinguishable at the single-cell level. Owing to the cell cycle-dependent expression of pRB a study of the proliferation status of the tumours was therefore conducted.

In gastric carcinomas of the diffuse type we observed significantly lower amounts of pRB and of PCNA than in intestinal and atypical carcinomas. Since previous reports (Saitoh *et al.*, 1992; David *et al.*, 1994) have shown that carcinomas of the diffuse type are less proliferative than intestinal-type carcinomas, our data suggest, although indirectly, that pRB immunoreactivity associates with cell proliferation in gastric cancer. This hypothesis is further supported by our flow cytometric evaluation of cell proliferation in these cases, showing a consistent trend (albeit not statistically significant) between cell proliferative indexes and pRB immunoreactivity scores (data not shown).

It is important to note, however, that PCNA scores probably overestimate the number of proliferative cells, since PCNA is detected in a very high percentage of cells. Previous reports (Hall *et al.*, 1990; Rosa *et al.*, 1992) suggest that PCNA immunoreactivity may be detected in cancer cells that have recently left the cycle owing to the long half-life of the antigen or to alteration of mRNA stability induced by growth factors.

It is tempting to speculate that the same or other epigenetic mechanisms affect pRB expression in gastric carcinomas, as a close relation was found between pRB and PCNA immunoreactivities. Alternatively, pRB may be identifying cell populations in earlier phases of the cell cycle than those identified by PCNA. This hypothesis would explain the cases in which higher immunoreactive scores were observed for pRB than for PCNA.

Western blot analysis revealed normal patterns in the 12 primary gastric carcinomas studied, thus excluding gross changes in pRB. The absence of phosphorylated forms in 'normal' mucosas is a previously well-documented phenomenon (Xu *et al.*, 1991) and occurs because the proliferative fraction (neck zone of the glands) is rather small when compared with the total number of cells and, therefore, undetectable by the technique used. The same explanation and/or stromal contamination may explain the absence of phosphorylated bands in the tumours.

The few studies published on chromosomal abnormalities in stomach cancer using cytogenetic analysis (Ochi *et al.*, 1986; Ferti-Passantonopoulou *et al.*, 1987; Seruca *et al.*, 1993) report, almost unanimously, that chromosome 13 is one of the most common chromosomes affected in these type of tumours. Studies conducted in order to establish the frequency of allele losses on chromosome 13 in primary gastric cancers, reported by Motomura *et al.* (1988) and Wada *et al.* (1988), revealed loss of heterozygosity in 41% and 11% of informative cases respectively. None of these studies, however, was designed to evaluate directly the allelic loss within the retinoblastoma gene.

In our study RB1 structure was checked by Southern blotting, using two genomic probes, and two cDNA probes in the 41 cases of gastric carcinomas. No obvious RB1 rearrangement or homozygous deletions were detected. Allelic imbalance was found in 29% of informative cases. Total loss of one allele was seen in two of the five cases showing allelic imbalance, thus suggesting that in a minor proportion of cases the RB1 gene may be important for the oncogenesis and/or progression of these tumours. On the other hand, the true nature of the remaining cases showing allelic imbalance is difficult to ascertain. They may be due to the result of clonal variation in RB1 copy number (duplication or loss) and/or stromal contamination.

In one case duplication of both alleles was detected. Monosomy of the chromosome 22 control probe was excluded since the filters were rehybridised with other VNTR probes (for chromosomes 6 and 17) and an equal loading in the normal and tumour lanes was confirmed. This finding is probably the result of an increase in chromosome copy number (tetrasomy) as duplication of both tumour alleles was also seen with a telomeric probe (p9A7) (data not shown).

In three cases (28, 30 and 36), all showing alterations with probe p68RS2.0, the 5.4 and 7.5 kb pG3.8M/*Hind*III fragments flanking this intronic region showed no change in intensity signal when compared with normal mucosa fragments. These findings are therefore suggestive that alterations were confined to this intronic region, which is keeping with the suggestion that the DNA sequence detected by probe p68RS2.0 might contain hotspots for structural aberrations (T'Ang *et al.*, 1989). Further studies are, however, required to confirm these findings.

It is noteworthy that no clear correlation was found between molecular alterations and loss of pRB immunoreactivity in our series. Similar findings have been described for other tumours in which RB1 allelic loss was not accompanied by loss of immunoreactivity (Ishikawa *et al.*, 1991; Borg *et al.*, 1992). Loss of pRB immunoreactivity also did not correlate with clinicopathological features such as tumour site, tumour staging, vascular invasion, nodal metastases, etc.

In summary, our results suggest that RB1 does not play, by itself, a crucial role in the oncogenesis of human gastric carcinomas. This contention is based on the following observations: pRB expression was found in all gastric carcinomas analysed and is associated with the proliferation status of the tumours; pRB protein, as assessed by Western blotting, showed normal patterns (110–116 kDa); loss of heterozygosity is not a frequent event and gross alterations such as homozygous deletions and or rearrangements were undetectable.

We acknowledge the gift of probes pGH2 and pG3.8M from Dr Y.-K.T. Fung and p68RS2.0 and p123M1.8 from Dr T. Dryja. We are grateful to Dr A. Wenzel for providing the RB1 and neuroblastoma cell lines. We gratefully acknowledge the valuable suggestions of Professor Sobrinho-Simões and Dr Hans Scheffer and the statistical analysis of the results by Professor Henrique Barros. We thank Dr Maria José Bento and Mrs Dina Leitão for technical assistance and Mr Abílio Ferreira for photographic assistance. This work was financially supported by Grant SAU 192/90-94 from the Junta Nacional de Investigação Científica e Tecnológica (JNICT).

### References

BÁRTEK, J., VOJTESEK, B., GRAND, R.J.A., GALLIMORE, P.H. & LANE, D.P. (1992). Cellular localization and T antigen binding of the retinoblastoma protein. *Oncogene*, **7**, 101-108.

BLANQUET, V., TURLEAU, C., GROUCHY, J. & CREAU-GOLDBERG, N. (1991). Physical map around the retinoblastoma gene: possible genomic imprinting suggested by NruI digestion. *Genomics*, **10**, 350-355.

BOOKSTEIN, R., PASCALE, R., MADREPERLA, S.A., HONG, F., ALLRED, C., GRIZZLE, W.E. & LEE, W.-H. (1990). Promoter deletion and loss of retinoblastoma gene expression in human prostate carcinoma. *Proc. Natl Acad. Sci. USA*, **87**, 7762-7766.

BORG, Å., ZHANG, Q.-X., ALM, P., OLSSON, H. & SELLBERG, G. (1992). The retinoblastoma gene in breast cancer: allele loss is not correlated with loss of gene protein expression. *Cancer Res.*, **52**, 2991-2944.

CAIRNS, P., PROCTOR, A.J. & KNOWLES, M.A. (1991). Loss of heterozygosity at the RB locus is frequent and correlates with muscle invasion in bladder carcinoma. *Oncogene*, **6**, 2305-2309.

CAVENEE, W.K., DRYJA, T.P., PHILLIPS, R.A., BENEDICT, W.F., GODBOUT, R., GALLIE, B.L., MURPHREE, A.L., STRONG, L.C. & WHYTE, R.L. (1983). Expression of recessive alleles by chromosomal mechanisms in retinoblastoma. *Nature*, **305**, 779-784.

DAVID, L., SANSONETTY, F., SOARES, P., CARNEIRO, F., AZEVEDO, R.M., LOPES, C. & SOBRINHO-SIMÕES, M. (1994). DNA content of gastric lesions with particular emphasis on gastric carcinoma. A study of 71 consecutive cases using flow cytometry. *Digest. Dis. Pathol.* (in press).

DE CAPRIO, J.A., LUDLOW, J.W., FIGGE, J., SHEW, J.-Y., HUANG, C.-M., LEE, W.-H., MARSILIO, E., PAUCHA, E. & LIVINGSTON, D.M. (1988). SV40 large tumor antigen forms a specific complex with the product of the retinoblastoma susceptibility gene. *Cell*, **54**, 275-283.

DRYJA, T.P., RAPAPORT, J.M., JOYCE, J.M. & PETERSEN, R.A. (1986). Molecular detection of deletions involving band q14 of chromosome 13 in retinoblastomas. *Proc. Natl Acad. Sci. USA*, **83**, 7391-7394.

FEINBERG, A.P. & VOGELSTEIN, B. (1983). A technique for radiolabeling DNA restriction endonuclease fragments to high specific activity. *Anal. Biochem.*, **132**, 6-13.

FERTI-PASSANTONOPOULOU, A.D., PANANI, A.D., VLACHOS, J.D. & RAPTIS, S.A. (1987). Common cytogenetic findings in gastric cancer. *Cancer Genet. Cytogenet.*, **24**, 63-73.

FRIEND, S.H., BERNARDS, R., ROGELJ, S., WEINBERG, R.A., RAPAPORT, J.M., ALBERT, D.M. & DRYJA, T.P. (1986). A human DNA segment with properties of the gene that predisposes to retinoblastoma and osteosarcoma. *Nature*, **323**, 643-646.

FRIEND, S.H., HOROWITZ, J.M., GERBER, M.R., WANG, X.-F., BOGENMANN, E., LI, F.P. & WEINBERG, R.A. (1987). Deletions of a DNA sequence in retinoblastomas and mesenchymal tumors: organization of the sequence and its encoded protein. *Proc. Natl Acad. Sci. USA*, **84**, 9059-9063.

FUNG, Y.-K.T., MURPHREE, A.L., TANG, A., QIAN, J., HINRICHS, S.H. & BENEDICT, W.F. (1987). Structural evidence for the authenticity of the human retinoblastoma gene. *Science*, **236**, 1657-1661.

FURUKAWA, Y., DECAPRIO, J.A., BELVIN, M. & GRIFFIN, J.D. (1991). Heterogeneous expression of the product of the retinoblastoma susceptibility gene in primary human leukaemia cells. *Oncogene*, **6**, 1343-1346.

HALL, P.A., LEVISON, D.A., WOODS, A.L., YU, C.C.-W., KELLOCK, D.B., WATKINS, J.A., BARNES, D.M., GILLETT, C.E., CAMPLEJOHN, R., DOVER, R., WASEEM, N.H. & LANE, D.P. (1990). Proliferation cell nuclear antigen (PCNA) immunolocalization in paraffin sections: an index of cell proliferation with evidence of deregulated expression in some neoplasms. *J. Pathol.*, **162**, 285-294.

HARBOUR, J.W., LAI, S.-L., WHANG-PENG, J., GAZDAR, A.F., MINNA, J.D. & KAYE, F.J. (1988). Abnormalities in structure and expression of the human retinoblastoma gene in SCLC. *Science*, **241**, 353-357.

HENSEL, C.H., HSIEH, C.-L., GAZDAR, A.F., JOHNSON, B.E., SAKAGUCHI, A.Y., NAYLOR, S.L., LEE, W.-H. & LEE, E.Y.-H.P. (1990). Altered structure and expression of the human retinoblastoma susceptibility gene in small cell lung cancer. *Cancer Res.*, **50**, 3067-3072.

HOROWITZ, J.M., YANDELL, D.W., PARK, S.-H., CANNING, S., WHYTE, P., BUCHKOVICH, K., HARLOW, E., WEINBERG, R.A. & DRYJA, T.P. (1989). Point mutational inactivation of the retinoblastoma antioncogene. *Science*, **243**, 937-940.

HSU, S.-M., RAINE, L. & FANGER, H. (1981). A comparative study of the peroxidase-antiperoxidase method and an avidin-biotin complex method for studying polypeptide hormones with radioimmunoassay antibodies. *Am. J. Clin. Pathol.*, **75**, 734-738.

ISHIKAWA, J., XU, H.-J., HU, S.-X., YANDELL, D.W., MAEDA, S., KAMIDONO, S., BENEDICT, W.F. & TAKAHASHI, R. (1991). Inactivation of the retinoblastoma gene in human bladder cancer and renal cell carcinomas. *Cancer Res.*, **51**, 5736-5743.

KNUDSON, A.G.J. (1971). Mutation and cancer: statistical study of retinoblastoma. *Proc. Natl Acad. Sci. USA*, **68**, 820-823.

LANDIS, J.R. & KOCH, G.G. (1977). The measurement of observer agreement for categorical data. *Biometrics*, **33**, 159-174.

LAURÉN, P. (1965). The two histological main types of gastric carcinoma: diffuse and so-called intestinal-type carcinoma. An attempt at a histoclinical classification. *Acta Pathol. Microbiol. Scand.*, **64**, 31-49.

LEE, W.-H., BOOKSTEIN, R., HONG, F., YOUNG, L.-J., SHEW, J.-Y. & LEE, E.Y.-H.P. (1987). Human retinoblastoma susceptibility gene: cloning, identification, and sequence. *Science*, **235**, 1394-1399.

LEE, E.H.-Y.P., TO, H., SHEW, J.-H., BOOKSTEIN, R., SCULLY, P. & LEE, W.-H. (1988). Inactivation of the retinoblastoma suceptibility gene in human breast cancers. *Science*, **241**, 218-221.

MARTÍNEZ, J.C., PIRIS, M.A., SÁNCHEZ-BEATO, M., VILLUENDAS, R., ORRADRE, J.L., ALGARA, P., SÁNCHEZ-VERDE, L. & MARTÍNEZ, P. (1993). Retinoblastoma (Rb) gene product expression in lymphomas. Correlation with Ki67 growth fraction. *J. Pathol.*, **169**, 405-412.

MOTOMURA, K., NISHISHO, I., TAKAI, S.-I., TATEISHI, H., OKAZAKI, M., YAMAMOTO, M., MIKI, T., HONJO, T. & MORI, T. (1988). Loss of alleles at loci on chromosome 13 in human primary gastric cancers. *Genomics*, **2**, 180-184.

MÜLLENBACH, R., LOGODA, P.J.L. & WELTER, C. (1989). An efficient salt chloroform extraction of DNA from blood and tissues. *Trends Genet.*, **5**, 391.

OCHI, H., DOUGLASS, H.O. & SANDBERG, A.A. (1986). Cytogenetic studies in primary gastric cancer. *Cancer Genet. Cytogenet.*, **22**, 295-307.

REISSMANN, P.T., SIMON, M.A., LEE, W.-H. & SLAMON, D.J. (1989). Studies of the retinoblastoma gene in human sarcomas. *Oncogene*, **4**, 839-843.

ROSA, J.C., MENDES, R., FILIPE, M.I. & MORRIS, R.W. (1992). Measurement of cell proliferation in gastric carcinoma: comparative analysis of Ki-67 and proliferative cell nuclear antigen (PCNA). *Histochem. J.*, **24**, 93-101.

SAITOH, K., CHIBA, T. & NAKAMURA, K. (1992). Cell proliferation kinetics of human gastric carcinoma: an immunohistochemical study with a monoclonal antibody against DN polymerase-alfa. *Eur. J. Cancer*, **28A**, **10**, 1642-1646.

SERUCA, R., CASTEDO, S., CORREIA, C., GOMES, P., CARNEIRO, F., SOARES, P., DE JONG, B. & SOBRINHO-SIMÕES, M. (1993). Cytogenetic findings in eleven gastric carcinomas. *Cancer Genet. Cytogenet.*, **68**, 42-48.

SHEW, J.-Y., LING, N., YANG, X., FODSTAD, O. & LEE, W.-H. (1989). Antibodies detecting abnormalities of the retinoblastoma susceptibility gene product (pp110RB) in osteosarcomas and synovial sarcomas. *Oncogene Res.*, **1**, 205-214.

TANG, A., KAI-JIN, W., HASHIMOTO, T., LIU, W.-Y., TAKAHASHI, R., SHI, X.-H., MIHARA, K., ZHANG, F.-H., CHEN, Y., DU, C., QIAN, J., LIN, Y.-G., MURPHREE, A.L., QIU, W.-R., THOMPSON, T., BENEDICT, W.F. & FUNG, Y.-K.T. (1989). Genomic organization of the human retinoblastoma gene. *Oncogene*, **4**, 401-407.

TOGUCHIDA, J., ISHIZAKI, K., SASAKI, M.S., IKENAGA, M., SUGIMOTO, M., KOTOURA, Y. & YAMAMURO, T. (1988). Chromosomal reorganization for the expression of recessive mutation of retinoblastoma susceptibility gene in the development of osteosarcoma. *Cancer Res.*, **48**, 3939–3943.

VARLEY, J.M., ARMOUR, J., SWALLOW, J.E., JEFFREYS, A.J., PONDER, B.A.J., TANG, A., FUNG, Y.-K.T., BRAMMAR, W.J. & WALKER, R.A. (1989). The retinoblastoma gene is frequently altered leading to loss of expression in primary breast tumors. *Oncogene*, **4**, 725–729.

WADA, M., YOKOTA, J., MIZOGUCHI, H., SUGIMURA, T. & TERADA, M. (1988). Infrequent loss of chromosomal heterozygosity in human stomach cancer. *Cancer Res.*, **48**, 2988–2992.

WIGGS, J., NORDENSKJÖLD, M., YANDELL, D., RAPAPORT, J., GRONDIN, V., JANSON, M., WERELIUS, B., PETERSEN, R., CRAFT, A., RIEDEL, K., LIBERFARB, R., WALTON, D., WILSON, W. & DRYJA, T.P. (1988). Prediction of the risk of hereditary retinoblastoma, using DNA polymorphisms within the retinoblastoma gene. *N. Engl. J. Med.*, **318**, 151–157.

XU, H.-J., HU, S.-X. & BENEDICT, W.F. (1991). Lack of nuclear RB protein staining in G0/middle G1 cells: correlation to changes in total RB protein level. *Oncogene*, **6**, 1139–1146.

YOKOTA, J., AKIYAMA, T., FUNG, Y.-K.T., BENEDICT, W.F., NAMBA, Y., HANOKA, M., WADA, M., TERSAKI, T., SHIMOSATO, Y., SUGIMURA, T. & TERADA, M. (1988). Altered expression of the retinoblastoma (RB) gene in small-cell carcinoma of the lung. *Oncogene*, **3**, 471–475.