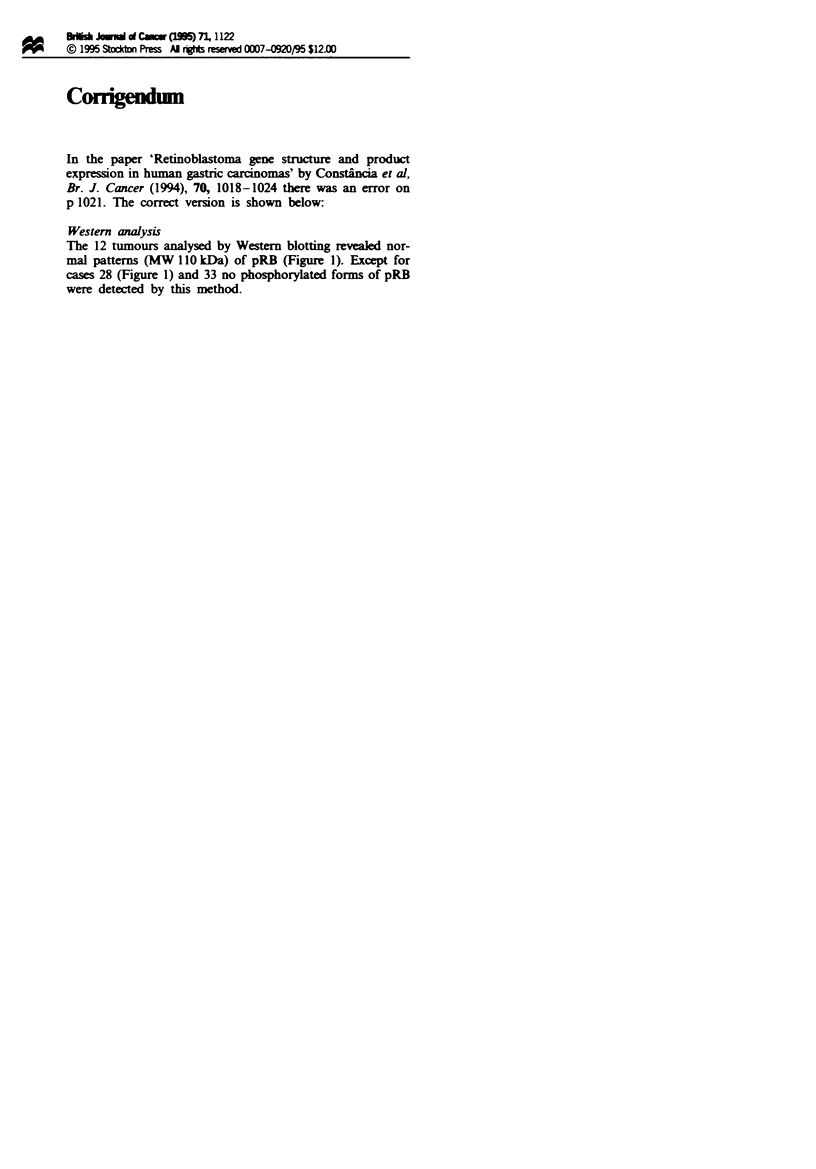# Corrigendum

**Published:** 1995-05

**Authors:** 


© 1995 Stockton Press All rights reserved 0007-0920/95 $12.00

### Corrigendum

In the paper 'Retinoblastoma gene structure and product expression in human gastric carcinomas' by Constância *et al*, *Br. J. Cancer* (1994), **70**, 1018-1024 there was an error on p 1021. The correct version is shown below:

*Western analysis*
The 12 tumours analysed by Western blotting revealed normal patterns (MW 110 kDa) of pRB (Figure 1). Except for cases 28 (Figure 1) and 33 no phosphorylated forms of pRB were detected by this method.